# Carrier Proteins Facilitate the Generation of Antipolysaccharide Immunity via Multiple Mechanisms

**DOI:** 10.1128/mbio.03790-21

**Published:** 2022-04-14

**Authors:** Fan Zhang, Claudette Thompson, Nicole Ma, Ying-Jie Lu, Richard Malley

**Affiliations:** a Department of Medicine, Division of Infectious Diseases, Boston Children’s Hospital, Harvard Medical School, Boston, Massachusetts, USA; Albert Einstein College of Medicine

**Keywords:** capsular polysaccharide, immune mechanisms, immunization

## Abstract

Capsular polysaccharides (CPSs) are important antigenic targets against bacterial infections. As T-independent antigens, however, CPSs elicit short-lived immune responses in adults and are poorly immunogenic in young children. Coupling CPS with protein carriers enhances anti-CPS responses and generates long-lasting immune memory. However, the mechanisms whereby carrier proteins accomplish this are not fully understood. Here, we dissect different mechanisms whereby carrier proteins enhance anti-CPS immunity. We show how coupling CPS with protein carriers modifies the interaction of CPS with antigen-presenting cells, enables a dual-activation mechanism for CPS-specific B cells via interaction with CPS- or carrier-specific T helper cells, and potentiates the recall of anti-CPS responses by engaging memory T helper cells during subsequent vaccination or bacterial exposure. Our findings provide new insights into the immunological basis of carrier-mediated anti-CPS immunity and may help in the design of more effective CPS-based vaccines.

## INTRODUCTION

Capsular polysaccharides (CPSs) are the main constituent of bacterial capsules and play important roles in maintaining bacterial structure, facilitating adherence to host cells, and preventing complement-mediated opsonic killing by phagocytes (1–3). Generating functional antibodies to CPS can protect against mucosal acquisition and invasive disease due to encapsulated bacterial pathogens (4–6). Therefore, CPSs have been used as important antigen targets for many bacterial vaccines. However, with a few exceptions (7, 8), most bacterial CPSs are type II T-independent (TI) antigens: they activate B cells (by cross-linking surface receptors) without engaging cognate T helper (Th) cells, leading to poor antibody production and no long-lived immune memory. As a consequence, immunization with vaccines comprised of purified CPS usually induces little to no anti-CPS responses in infants or young children and only transient antibody production in adults, even when given at a high dose (e.g., 25 μg or higher per CPS).

The development of polysaccharide (PS)-protein conjugate vaccines, comprised of CPS covalently linked to protein carriers (9), has overcome this problem. Conjugate vaccines induce robust anti-CPS responses in infants and provide effective protection against invasive disease caused by encapsulated bacterial pathogens, including Streptococcus pneumoniae, Haemophilus influenzae type b (Hib), Neisseria meningitidis types A, C, W135, and Y, and, most recently, Salmonella enterica serovar Typhimurium (10–13). Studies then revealed important immunological properties of conjugate-induced anti-CPS responses, including Ig class switching (from IgM to IgG), Ig affinity maturation, major histocompatibility complex class II (MHCII) dependency, and immune memory generation (8, 14–17), indicating that CPS-protein conjugates, in contrast to pure CPSs, can activate CPS-specific B cells (B_CPS_) via a classical T-dependent (TD) pathway.

Following the success of polysaccharide-protein conjugates, other CPS-protein vaccines (using different types of association between CPS and protein carriers) have been proposed and developed. One platform uses a protein matrix onto which the CPS is nonspecifically absorbed: in preclinical studies, such a construct was shown to induce IgG antibodies, a feature of TD responses, to two studied CPS antigens (18). Our group developed another approach, called MAPS (for multiple-antigen-presenting system), in which CPSs are biotinylated and tightly coupled (dissociation constant [*K_d_*] of ∼10^−15^ M) to pathogen-specific proteins to which an avidin-like protein (rhizavidin [rhavi]) is genetically fused (19, 20). We showed that MAPS can induce the same TD anti-CPS responses at a magnitude comparable, and at times superior, to those obtained with CPS-protein conjugates (20, 21). Most recently, a MAPS vaccine at a dose of 1, 2, or 5 μg of each of 24 pneumococcal polysaccharides coupled to a rhavi-fusion of pneumococccal proteins was shown to generate robust functional anti-CPS IgG antibodies in healthy young and older adults, with immunogenicity comparable (and, in some cases, superior) to that of the licensed 13-valent pneumococcal vaccine, Prevnar 13, for the common serotypes (22).

In this work, using MAPS, we explored the mechanisms that mediate such a TI-to-TD switch of anti-CPS responses with CPS-protein vaccines. We sought to examine whether the mechanism varies depending on the individual CPS or the type of interaction between CPS and proteins. Our results indicate that carrier proteins can facilitate TD anti-CPS responses via three separate mechanisms. First, we show that associations with carrier proteins modify the binding, internalization, processing, and presentation of CPS by antigen-presenting cells (APCs), the first and necessary step for acquired immune responses. Furthermore, we show that the type of CPS-protein association determines whether the CPS will be presented in an MHCII-dependent or -independent manner. Next, we show that during priming (primary vaccination), coupling with carrier proteins enables a dual-activation mechanism for naive B_CPS_, via interaction with CPS-specific T helper cells (T_CPS_) or carrier-specific T helper cells (T_carrier_). The proportion of B_CPS_ activation via each route may vary depending on the individual CPS antigens (in a given type of CPS-protein construct). Finally, we show that during recall, either by subsequent vaccination or by exposure to the bacterium, association with (pathogen-specific) carrier proteins can potentiate anti-CPS IgG production by memory B_CPS_, via engaging memory T_CPS_ and/or T_carrier_.

## RESULTS

### Immunization with MAPS vaccines induces a TD anti-CPS response and immune memory.

**FIG 1:**
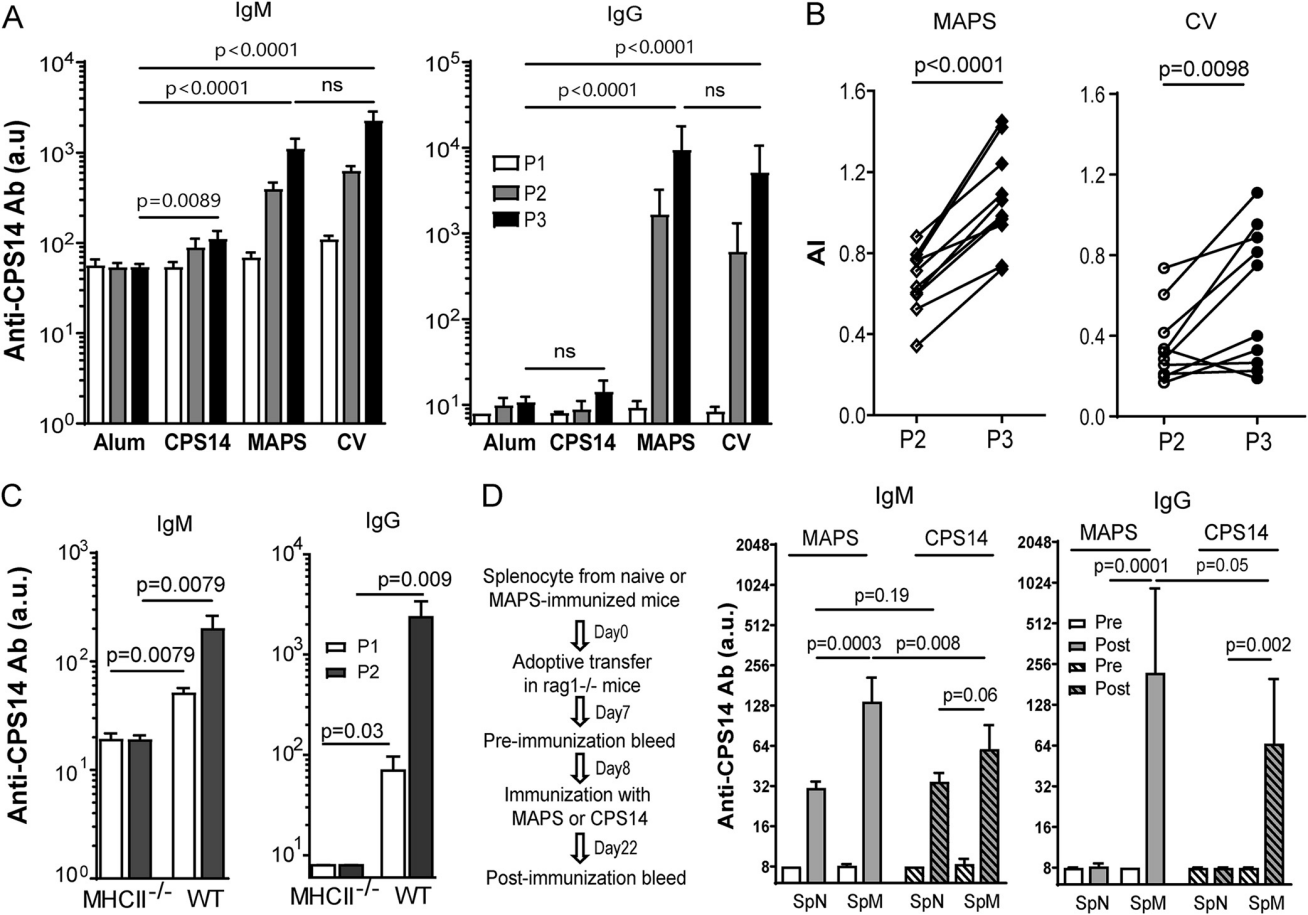
Immunization with MAPS vaccines induces TD anti-CPS responses and immune memory. (A and B) C57BL/6 mice (*n* = 10 per group) received three subcutaneous immunizations with adjuvant alone (alum), the adjuvanted CPS14 or CPS14 MAPS vaccine, or the CPS14 conjugate vaccine (CV) (1 μg of CPS content per dose). (A) Anti-CPS IgM and IgG antibodies (Ab) in each group after one (P1), two (P2), or three (P3) immunizations. ns, not significant. (B) Avidity of anti-CPS IgG antibodies in CPS14 MAPS-immunized mice after two (P2) or three (P3) immunizations. AI, avidity index. (C) Anti-CPS IgM and IgG antibodies in wild-type (WT) or MHCII^−/−^ C57BL/6 mice (*n* = 5 per group) after one (P1) or two (P2) immunizations with the CPS14 MAPS vaccine. (D) Rag1^−/−^ mice (*n* = 8 to 10 per group) received an adoptive transfer of splenocytes isolated from naive mice (SpN) or from CPS14 MAPS-immunized mice (SpM). Eight days after adoptive transfer, Rag1^−/−^ mice received one immunization with uncoupled CPS14 (CPS14) or CPS14 MAPS (MAPS) (1 μg of PS content per dose). Anti-CPS IgG antibodies were measured 1 day before (Pre) and 14 days after (Post) immunization. For all panels, antibody titers are expressed in arbitrary units (a.u.) relative to a reference serum sample for CPS14 antigen (see Materials and Methods). Bars represent geometric means and 95% confidence intervals (CIs). Statistical analyses were performed using the Mann-Whitney U test, in comparison to the alum group (A) or as indicated (B to D).

We previously demonstrated that MAPS vaccines induce robust anti-CPS IgG antibodies in a CD4^+^ T cell-dependent manner (20). Here, we further characterize the properties of MAPS-induced TD anti-CPS responses and immune memory. C57BL/6 mice were immunized with either type 14 pneumococcal CPS (CPS14), a MAPS vaccine consisting of biotinylated CPS14 coupled to avidin protein (Table 1), or a conjugate vaccine (CV) consisting of CPS14 conjugated to tetanus toxoid, all adjuvanted with aluminum phosphate (alum) or with alum alone (negative control). Anti-CPS14 IgM and IgG antibodies were measured 2 weeks after each immunization. Like human infants, adult mice do not respond well to pure CPS antigens: immunization with CPS14 resulted in a low level of anti-CPS IgM but no IgG antibodies due to the TI activation of B_CPS_ (Fig. 1A, CPS14). In contrast, immunization with either the MAPS vaccine or the conjugate vaccine induced high levels of IgM and IgG anti-PS antibodies (Fig. 1A, MAPS and CV) and was associated with antibody affinity maturation (Fig. 1B). As shown in Fig. 1C, MAPS-induced anti-CPS responses are MHCII dependent, confirming the involvement of classical Th cells rather than other types of T cells (e.g., NKT cells).

**TABLE 1:** MAPS complexes used in this study

MAPS	CPS	Carrier protein
CPS14 MAPS	CPS14	Avidin

5V-MAPS1	CPS1	Rhizavidin-SP1500-SP0785 (carrier protein 1)
	CPS3	
	CPS4	
	CPS5	
	CPS14	

5V-MAPS2	CPS1	Avidin (carrier protein 2)
	CPS3	
	CPS4	
	CPS5	
	CPS14	

CPS4 MAPS	CPS4	Rhizavidin-PdT

An important outcome of TD immune responses is the generation of antigen-specific memory cells. The induction of CPS-specific memory cells by the MAPS vaccine was evaluated using adoptive cell transfer experiments. Four groups of Rag1^−/−^ mice received adoptive transfer of splenocytes isolated from naive (SpN) or MAPS-immunized (SpM) mice. One week later, Rag1^−/−^ mice received one immunization with either CPS14 or CPS14 MAPS. Anti-CPS IgM and IgG antibodies were measured 2 weeks after immunization. As shown in Fig. 1D, in Rag1^−/−^ mice that received naive splenocytes, one immunization with CPS14 (SpN, striped bars) or CPS14 MAPS (SpN, open bars) induced typical primary responses characterized by low concentrations of anti-CPS IgM and no detectable IgG antibodies. In contrast, in Rag1^−/−^ mice that received splenocytes from MAPS-immunized mice, one immunization with CPS14 MAPS led to increased anti-CPS IgM and, more importantly, robust production of anti-CPS IgG antibodies (Fig. 1D, SpM, open bars), reflecting recall responses in the presence of CPS-specific memory cells. Similar recall responses, associated with both anti-CPS IgM and IgG production, were also observed when SpM mice were immunized with CPS14 alone (Fig. 1D, SpM, striped bars) albeit at lower levels than those following immunization with MAPS. Thus, these results indicate that immunization with the MAPS vaccine induces anti-CPS memory cells that can be reactivated (recalled) by either pure CPS (i.e., in a TI manner) or MAPS vaccines (i.e., in a TD manner), resulting in the production of a high level of anti-CPS IgG antibody. The difference between TI and TD recall responses is further examined below.

### Coupling CPS with carrier proteins enhances uptake, processing, and surface presentation of CPS antigens in APCs.

**FIG 2:**
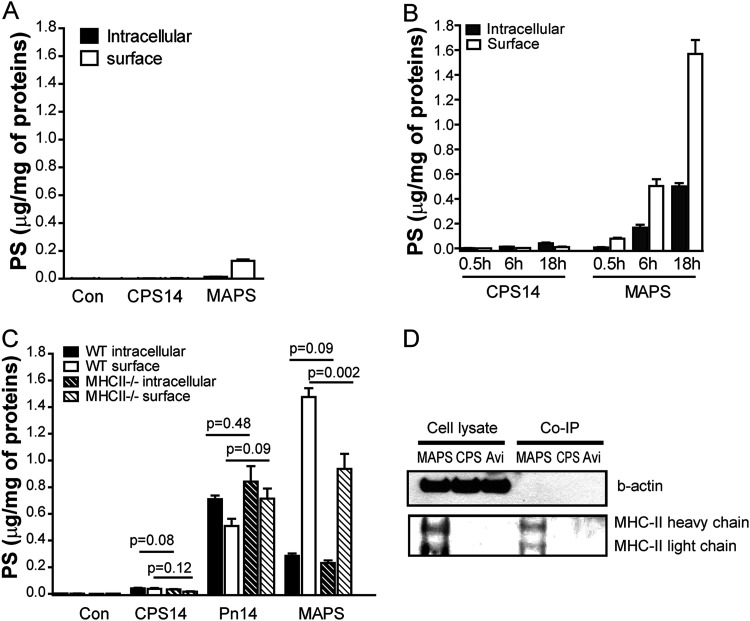
Coupling with carrier proteins enhances the uptake, processing, and MHCII-dependent and -independent surface presentation of CPS antigens in macrophages. (A and B) Peritoneal macrophages isolated from C57BL/6 mice were incubated in culture medium containing no CPS (control [Con]), 2.5 μg/mL of CPS14, or CPS14 MAPS (at 2.5 μg/mL of CPS content) at 4°C for 2 h (A) or at 37°C for the indicated periods (B). Intracellular and surface-associated CPS contents in different samples were measured by an inhibition ELISA and then normalized to the total cellular protein content (micrograms of CPS per milligram of protein). Bars represent means and standard errors of the means (SEM) (*n* = 12 in 4 independent experiments). (C) Internalization and presentation of purified CPS14, CPS14 in bacterial cells, or the MAPS complex in wild-type (WT) or MHCII^−/−^ macrophages. Peritoneal macrophages isolated from either WT or MHCII^−/−^ C57BL/6 mice were incubated in culture medium containing CPS14, heat-killed type 14 pneumococci (Pn14), or CPS14 MAPS (all at 2.5 μg/mL of CPS content) at 37°C for 18 h. Intracellular and surface-associated CPS contents in different samples were measured by an inhibition ELISA and then normalized to the total cellular protein content. Bars represent means and SEM (*n* = 9 in 3 independent experiments). Statistical analyses were performed using the Mann-Whitney U test between the indicated groups. (D) Peritoneal macrophages were incubated with CPS14 MAPS (2.5 μg/mL of CPS and 7.5 μg/mL of avidin), CPS14 (2.5 μg/mL), or avidin (Avi) (7.5 μg/mL) at 37°C for 18 h. After incubation, cells were washed with PBS twice and then lysed with lysis buffer. All cell lysates were then normalized by the total protein content measured by a BCA assay. For coimmunoprecipitation (Co-IP), each cell lysate was mixed with rabbit anti-CPS14 serum-pretreated protein A resins and incubated overnight at 4°C. After extensive washing with lysis buffer, the resins were boiled in SDS sample buffer, and the supernatants were then applied onto an SDS-PAGE gel. Western blotting was done using primary antibodies against β-actin (internal control) and MHCII.

To explore the mechanisms underlying MAPS-induced TD anti-CPS immunity, we first examined in APCs the uptake, processing, and presentation of CPS antigens, with or without coupling to carrier proteins. Peritoneal macrophages isolated from C57BL/6 mice were used for all assays. The binding and internalization of CPS antigens were examined after incubation with cells at 4°C or 37°C for various periods. The amount of CPS (in micrograms) present on the surface or inside the cells was measured by an inhibition enzyme-linked immunosorbent assay (ELISA) and then normalized to the total protein content of the cell lysates (per milligram). First, we noticed that uncoupled CPS14 (nonbiotinylated) had very little interaction with macrophages: there was minimum binding at 4°C and barely detectable internalization (<0.1 μg) after overnight incubation at 37°C (Fig. 2A and B, CPS14). In contrast, affinity coupling to avidin (as a MAPS complex) greatly enhanced the binding (∼0.16 μg) (Fig. 2A, MAPS) and internalization of CPS14 in macrophages (Fig. 2B). Prolonged incubation at 37°C resulted in significant increases in both intracellular CPS (as a result of internalization) (Fig. 2B, MAPS, gray bars) and surface-associated CPS (Fig. 2B, MAPS, open bars). At the end of the 6- or 18-h incubation, the level of surface-associated CPS was 0.5 or 1.6 μg, respectively, which far exceeded the level of surface binding of CPS (in MAPS) measured at 4°C or after a 0.5-h incubation at 37°C (<0.2 μg). Thus, we inferred that this increase may reflect the surface presentation of CPS (epitopes) after the intracellular processing of the internalized MAPS complexes.

To further characterize this surface presentation of CPS, we examined and compared the interactions of CPS14 MAPS with wild-type (WT) or MHCII^−/−^ macrophages. Also, in this experiment, we included heat-killed type 14 pneumococci (Pn14) as a comparison. While surrounded by many bacterial proteins, in Pn14, the CPS is covalently attached to peptidoglycan and has no direct connection to proteins (23). As expected, purified CPS14 (nonbiotinylated) alone had minimal interaction with either type of macrophage (Fig. 2C, CPS14). In contrast, in the context of bacterial cells (proteins), a significant amount of Pn14-CPS was captured by WT macrophages after overnight incubation. Over half of the CPS (∼0.7 μg) accumulated inside the cells (Fig. 2C, Pn14, black bar), and the rest (∼0.5 μg) was presented at the surface (after intracellular processing) (Fig. 2C, Pn14, white bar). The absence of MHCII molecules did not reduce the surface presentation of Pn14-CPS (Fig. 2C, Pn14, striped bars), suggesting that this presentation is mediated via other molecules, possibly CD1d (24, 25). In the case of CPS14 MAPS, overnight incubation led to ∼1.8 μg of total CPS associated with macrophages. Over 80% of this CPS (∼1.5 μg) was localized on the cell surface (Fig. 2C, MAPS, white bar), suggesting the more efficient processing/presentation of MAPS-CPS than Pn14-CPS. When incubated with MHCII^−/−^ macrophages, the surface presentation of MAPS-CPS was reduced by ∼40% compared to that in WT macrophages (Fig. 2C, MAPS, white bar versus white striped bar). The alternative, MHCII-independent presentation of MAPS-CPS in MHCII^−/−^ macrophages was comparable to the presentation of Pn14-CPS in either WT or MHCII^−/−^ macrophages (Fig. 2C, MAPS, striped white bar, versus Pn14, white and striped white bars). The association of MAPS-CPS14 with MHCII molecules in WT macrophages was further confirmed by coimmunoprecipitation using anti-CPS14 antibodies followed by Western blotting using anti-MHCII antibodies (Fig. 1D).

Thus, our data suggest that either directly coupling CPS with carrier proteins (as in MAPS complexes) or indirectly associating CPS with proteins (as in bacterial cells) can significantly enhance the uptake, intracellular processing, and presentation of CPS antigens in APCs. However, only in the case of MAPS complexes, where CPS is directly/tightly coupled to carrier proteins, is the processed CPS presented via MHCII molecules.

### Coupling with carrier proteins enables a dual-activation mechanism for naive B_CPS_ mediated by T_CPS_ and T_carrier_.

The presentation of antigens by MHCII leads to the activation of cognate Th cells. In the case of MAPS complexes, where CPS and protein carriers are coupled, APCs can present the CPS (as shown above) and the carrier protein simultaneously (as indicated by the robust anti-carrier IgG production following vaccination with MAPS [20, 26] [see Fig. S1 in the supplemental material]). This dual presentation could thus activate two different populations of Th cells, T_CPS_ and T_carrier_. B_CPS_, as a special type of APC, could then interact with T_CPS_ or T_carrier_ via the MHCII-presented CPS or carrier, respectively.

10.1128/mbio.03790-21.2 FIG S1 Immunization of mice with carrier 1 protein or 5V-MAPS1 generates robust anti-carrier 1 antibody responses. C57BL/6 mice (*n* = 10 per group) received no immunization (naive) or one immunization with carrier 1 or 5V-MAPS1, each at 15 μg of carrier 1 content per mouse. Anti-carrier 1 IgG antibodies were measured 14 days later. Antibody titers are expressed in arbitrary units (a.u.) relative to a reference serum sample for carrier 1. Bars represent geometric means and 95% CIs. Statistical analyses were performed using the Mann-Whitney U test compared to the naive group or between the carrier 1 and 5V-MAPS1 groups. Download FIG S1, TIF file, 1.7 MB. Copyright © 2022 Zhang et al. 2022 Zhang et al. https://creativecommons.org/licenses/by/4.0/ This content is distributed under the terms of the Creative Commons Attribution 4.0 International license.

Next, we sought to understand the contribution of these two types of Th cells to the activation of naive B_CPS_ during immunization with MAPS. Furthermore, we investigated whether the activation mechanism is the same or different for different CPS antigens.

**FIG 3:**
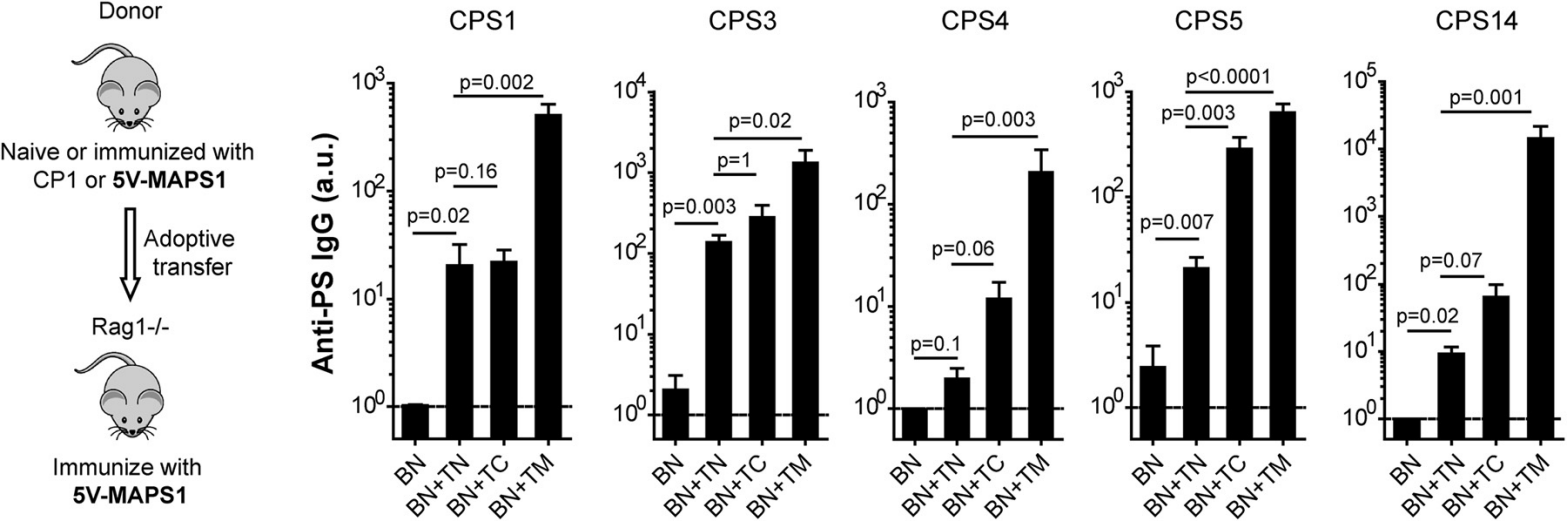
T_carrier_-mediated activation of naive B_CPS_ during MAPS vaccination. Rag1^−/−^ mice (*n* = 5 for group BN and *n* = 10 for the other groups) received an adoptive transfer of B cells isolated from naive mice (BN), alone or in combination with CD4^+^ T cells isolated from naive mice (TN), carrier 1-primed mice (TC), or 5V-MAPS1-primed mice (TM). Eight days later, Rag1^−/−^ mice received one immunization with 5V-MAPS1 (1 μg per CPS). Anti-CPS IgG antibodies were measured 1 day before (preimmunization) and 14 days after immunization. Antibody titers are expressed in arbitrary units (a.u.) relative to a reference serum sample for each CPS antigen. Dashed lines indicate geometric means of anti-CPS IgG titers of all groups preimmunization. Bars represent geometric means and 95% CIs of anti-CPS IgG titers of each group postimmunization. Statistical analyses were performed using the Mann-Whitney U test between the indicated groups.

We studied the role of individual Th cell populations in the activation of naive B_CPS_ by adoptive transfer. As the frequency of T_CPS_ or T_carrier_ is very low in naive mice, we enriched these Th cells by immunizing mice with one dose of MAPS vaccine or carrier protein. The role of T_carrier_ was evaluated as follows. A 5-valent MAPS vaccine (5V-MAPS1) was made by coupling five biotinylated pneumococcal CPSs, individually, with a carrier protein consisting of rhizavidin fused to two pneumococcal proteins, SP1500 and SP0785 (carrier 1) (Table 1). We immunized two groups of donor mice with either 5V-MAPS1 (to enrich both T_CPS_ and T_carrier_) or just carrier 1 (to enrich T_carrier_). Six weeks later, B cells or CD4^+^ T cells from naive (TN), carrier 1-immunized (TC), or 5V-MAPS1-immunized (TM) mice were isolated, and combinations of cells were adoptively transferred into Rag1^−/−^ mice as shown in Fig. 3. One week later, Rag1^−/−^ mice received one immunization with 5V-MAPS1. Preimmunization sera of Rag1^−/−^ mice showed no detectable anti-CPS IgG antibodies (Fig. 3, dashed line). Two weeks after vaccination, all Rag1^−/−^ mice that received a combination of naive B cells (BN) and a source of CD4^+^ T cells, but not mice that received BN alone, developed anti-CPS IgG antibodies (Fig. 3), reflecting TD activation of naive B_CPS_ by the MAPS vaccine. For all five CPS antigens, the group that received MAPS-primed Th cells (with enriched T_CPS_ and T_carrier_) had the most effective activation of B_CPS_ and produced the largest amount of anti-CPS IgG (Fig. 3, BN+TM). In contrast, transferring carrier 1-primed Th cells (with enriched T_carrier1_) had a differential effect on the antibody response to various CPS antigens. For CPS5 or, to a lesser extent, CPS4 and CPS14, there was a clear enhancement of anti-CPS IgG production in the presence of carrier 1-primed Th cells compared to naive Th cells; for CPS1 and CPS3, in contrast, the difference between the two groups was less obvious (Fig. 3, BN+TN versus BN+TC). This result therefore suggests that T_carrier_ can indeed mediate the activation of naive B_CPS_ but that this mechanism is more relevant for some CPS antigens than others. Furthermore, the greater activation of B_CPS_ to all five CPS antigens mediated by MAPS-primed Th cells suggests T_carrier_-independent activation mechanisms, likely mediated by T_CPS_.

**FIG 4:**
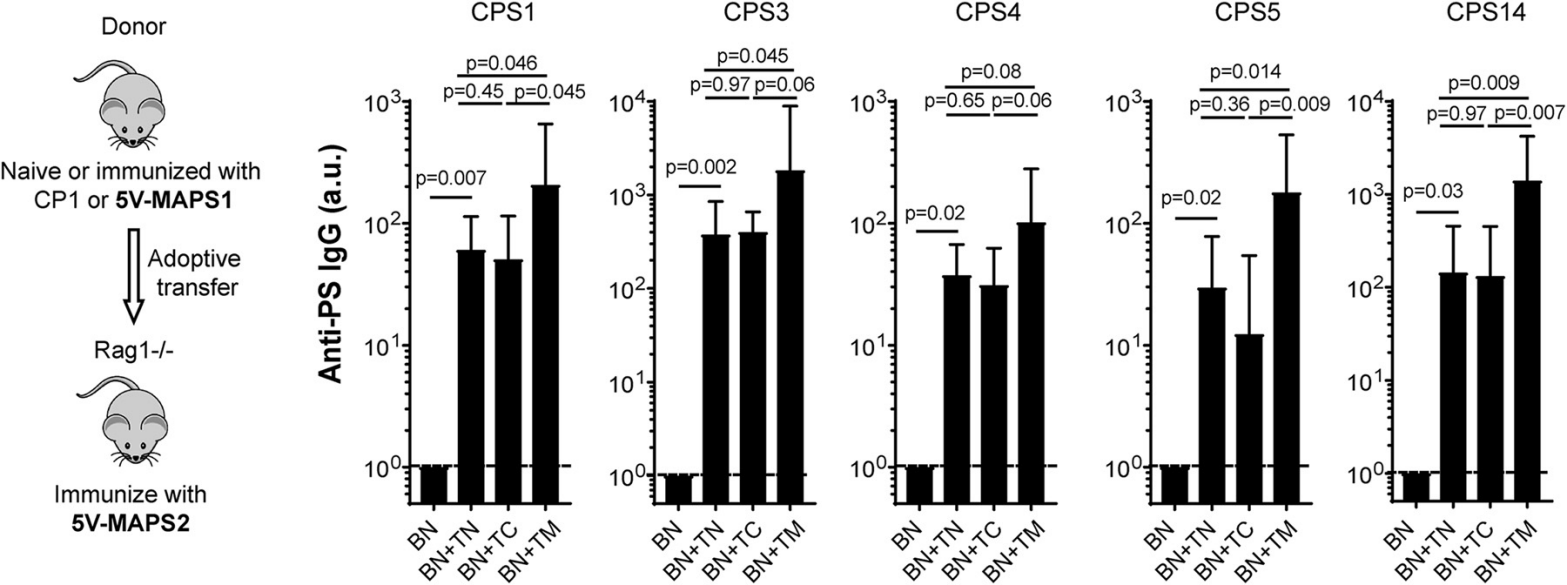
T_CPS_-mediated activation of naive B_CPS_ during MAPS vaccination. Rag1^−/−^ mice (*n* = 5 for group BN and *n* = 10 for the other groups) received an adoptive transfer of B cells isolated from naive mice (BN), alone or in combination with CD4^+^ T cells isolated from naive mice (TN), carrier 1-primed mice (TC), or 5V-MAPS1-primed mice (TM). Eight days after adoptive transfer, Rag1^−/−^ mice received one immunization with 5V-MAPS2 (2.5 μg per CPS). Anti-CPS IgG antibodies were measured 1 day before (preimmunization) and 14 days after immunization. Antibody titers are expressed in arbitrary units (a.u.) relative to a reference serum sample for each CPS antigen. Dashed lines indicate geometric means of anti-CPS IgG titers of all groups preimmunization. Bars represent geometric means and 95% CIs of anti-CPS IgG titers of each group postimmunization. Statistical analyses were performed using the Mann-Whitney U test between the indicated groups.

To further evaluate the contribution of T_CPS_ to B_CPS_ activation, Rag1^−/−^ mice received a transfer of naive B cells, alone or combined with naive, carrier 1-primed, or 5V-MAPS1-primed Th cells, as described above for the first experiment but were then immunized with 5V-MAPS2, a MAPS vaccine made with the same five CPS antigens but coupled to a different carrier protein, egg avidin (carrier 2) (Table 1). Carrier 2 and carrier 1 share no cross-reactive epitopes (Fig. S2 and S3). Therefore, the presence of enriched T_carrier_ to carrier 1 should not provide additional help to B cell activation following immunization with 5V-MAPS2. Consistent with this prediction, following 5V-MAPS2 vaccination, there was no increase in anti-carrier 2 or anti-CPS IgG production in Rag1^−/−^ mice that received carrier 1-primed Th cells compared to those that received naive Th cells (Fig. 4, BN+TN versus BN+TC, and Fig. S3). In contrast, transferring 5V-MAPS1-primed Th cells enhanced IgG production for all five CPS antigens compared to naive or carrier 1 Th cells (Fig. 4, BN+TM versus BN+TN or BN+TC). As the five CPSs are the only antigens in common between 5V-MAPS1 and 5V-MAPS2, this enhanced anti-CPS IgG production likely reflects the contribution of T_CPS_ (enriched in 5V-MAPS1-primed Th cells) to B_CPS_ activation. Overall, our results indicate that following immunization with MAPS, naive B_CPS_ can be activated via two independent Th cell populations, T_CPS_ or T_carrier_, the relative contribution of which may depend on the individual CPS antigen.

10.1128/mbio.03790-21.3 FIG S2 Rhavi-specific Th2 cells do not cross-react with egg avidin. C57BL/6 mice received either no immunization (naive) (*n* = 5) or three immunizations with rhavi (*n* = 10) at 5 μg per mouse. Antigen-specific Th2 responses were examined by interleukin-5 (IL-5) production after *ex vivo* stimulation of peripheral blood samples of immunized mice with rhavi or egg avidin (both at 10 μg/mL in Dulbecco’s modified Eagle’s medium [DMEM]–F-12 medium containing 10% low-endotoxin defined fetal bovine serum [FBS] [HyClone], 50 μM 2-mercaptoethanol [Sigma], and ciprofloxacin [10 μg/mL; Cellgro]). After incubation at 37°C for 6 days, the culture supernatants were collected, and the concentration of IL-5 in each sample was measured using an IL-5 ELISA kit (BioLegend). Rhavi, but not egg avidin, activated Th2 cells of rhavi-immunized mice, leading to IL-5 production. Download FIG S2, TIF file, 1.7 MB. Copyright © 2022 Zhang et al. 2022 Zhang et al. https://creativecommons.org/licenses/by/4.0/ This content is distributed under the terms of the Creative Commons Attribution 4.0 International license.

10.1128/mbio.03790-21.4 FIG S3 The presence of carrier 1-primed or 5V-MAPS1-primed Th cells does not enhance anti-carrier 2 IgG production during 5V-MAPS2 vaccination. Rag1^−/−^ mice (*n* = 10 per group) received an adoptive transfer of B cells purified from naive mice (BN), alone or in combination with CD4^+^ T cells purified from naive mice (TN), carrier 1-primed mice (TC), or 5V-MAPS1-primed mice (TM), as described in the legend of Fig. 4. Eight days after adoptive transfer, Rag1^−/−^ mice received one immunization with 5V-MAPS2. Anti-carrier 1 and anti-carrier 2 IgG antibodies were measured 14 days after immunization. Antibody titers are expressed in arbitrary units (a.u.) relative to a reference serum sample for carrier 1 or carrier 2. Bars represent geometric means and 95% CIs. Statistical analyses were performed using the Mann-Whitney U test. n.s, not significant. Download FIG S3, TIF file, 2.3 MB. Copyright © 2022 Zhang et al. 2022 Zhang et al. https://creativecommons.org/licenses/by/4.0/ This content is distributed under the terms of the Creative Commons Attribution 4.0 International license.

### Memory Th cells potentiate the recall of anti-CPS responses mediated by memory B_CPS_.

A direct outcome of TD activation of naive B_CPS_ is the generation of memory B_CPS_, which, upon reexposure to CPS antigen, can rapidly upregulate IgG antibody production (a process often referred to as a recall response). In contrast to primary responses, recall anti-CPS responses do not have to be induced by TD CPS antigens (e.g., CPS-protein conjugates or complexes). Clinically, recall anti-CPS responses have been seen when previously (conjugate vaccine) immunized individuals are boosted with pure CPS (e.g., Hib CPS or 23-valent pneumococcal CPS vaccine). Our data here showed the same phenomenon in mice: a recall anti-CPS14 response is observed following the injection of pure CPS14 in mice that received an adoptive transfer of MAPS-primed splenocytes (Fig. 1D). However, we also noticed that the CPS14-induced recall response is of a lower magnitude than that of the recall response following exposure to CPS14 MAPS (a TD antigen) at the same dosage (1 μg of CPS) (Fig. 1D).

**FIG 5:**
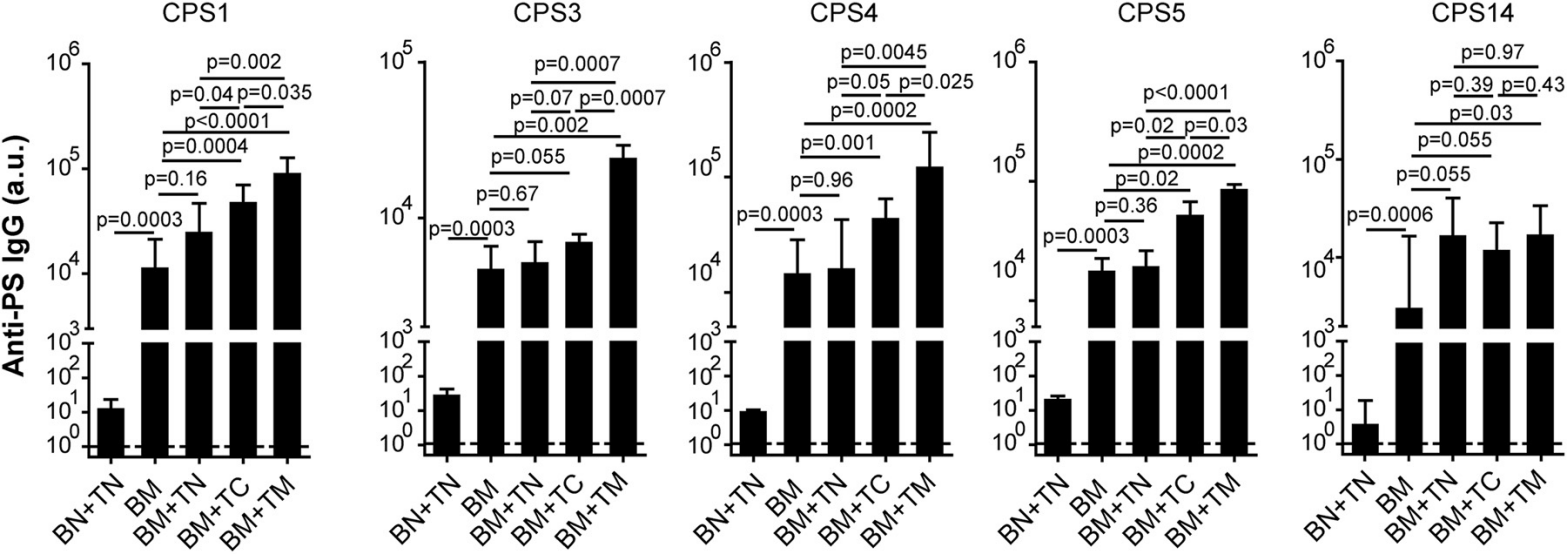
Anti-CPS recall responses in the absence or presence of naive, carrier-primed, or MAPS-primed CD4^+^ T cells. Rag1^−/−^ mice (*n* = 7 to 10 per group) received an adoptive transfer of B and CD4^+^ T cells isolated from naive mice (BN+TN) or B cells isolated from 5V-MAPS1-primed mice (BM), alone or in combination with CD4^+^ T cells isolated from naive mice (TN), carrier 1-primed mice (TC), or 5V-MAPS1-primed mice (TM). Eight days after adoptive transfer, Rag1^−/−^ mice received one immunization with 5V-MAPS1 (1 μg per CPS content). Anti-CPS IgG antibodies were measured 1 day before (preimmunization) and 14 days after immunization. Antibody titers are expressed in arbitrary units (a.u.) relative to a reference serum sample for each CPS antigen. Dashed lines indicate geometric means of anti-CPS IgG titers of all groups preimmunization. Bars represent geometric means and 95% CIs of anti-CPS IgG titers of each group postimmunization. Statistical analyses were performed using the Mann-Whitney U test between the indicated groups.

To explore the mechanisms underlying the differential anti-CPS recall responses to TI versus TD CPS antigens, we studied the role of various Th cells in memory B_CPS_ activation. We isolated B cells from 5V-MAPS1-immunized mice (BM) as a source of memory B_CPS_ and adoptively transferred BM cells, alone or in combination with naive (TN), carrier 1-primed (TC), or 5V-MAPS1-primed (TM) Th cells, into Rag1^−/−^ mice. A separate group received naive B and Th cells (BN+TN) to provide a primary response baseline. One week after adoptive transfer, Rag1^−/−^ mice received one immunization with 5V-MAPS1. No anti-CPS IgG was detected in preimmunization sera (Fig. 5, dashed lines). Two weeks after immunization, mice in all groups that received BM cells (containing memory B_CPS_), with or without Th cells, displayed robust recall responses, with anti-CPS IgG titers being up to a thousandfold higher than the primary response mediated by naive B and Th cells (Fig. 5). Interestingly, we found that for four out of five CPS antigens (except for CPS14), the presence of naive Th cells provided no significant help to the activation of memory B_CPS_: the level of anti-CPS IgG production was comparable to that with the TI recall responses seen in the BM group (Fig. 5, BM+TN versus BM). In contrast, the presence of carrier 1-primed (with memory T_carrier_) or, even more so, MAPS-primed (with memory T_CPS_ and T_carrier_) Th cells could significantly enhance recall anti-CPS IgG production by memory B_CPS_ for these four CPS antigens (Fig. 5, BM+TC or BM+TM versus BM). In the case of CPS14, it appeared that naive Th cells alone were sufficient to enhance recall responses. Together, these results confirm that memory B_CPS_ can indeed be activated in a TI manner and also indicate that a TD antigen (i.e., a MAPS complex) can induce enhanced recall anti-CPS responses, in most cases, via engaging memory (rather than naive) T_carrier_ and/or T_CPS_.

### A pathogen-specific carrier protein enhances anti-CPS recall responses upon pathogen exposure.

Our results described above revealed an important role of memory Th cells, especially memory T_carrier_, in enhancing anti-CPS recall responses. This finding thus raises the intriguing possibility that immunization with a CPS vaccine comprising a protein carrier derived from the target pathogen could induce memory T_carrier_ that can be triggered during infections by the endogenous bacterial protein and thus facilitate stronger anti-CPS recall responses.

**FIG 6:**
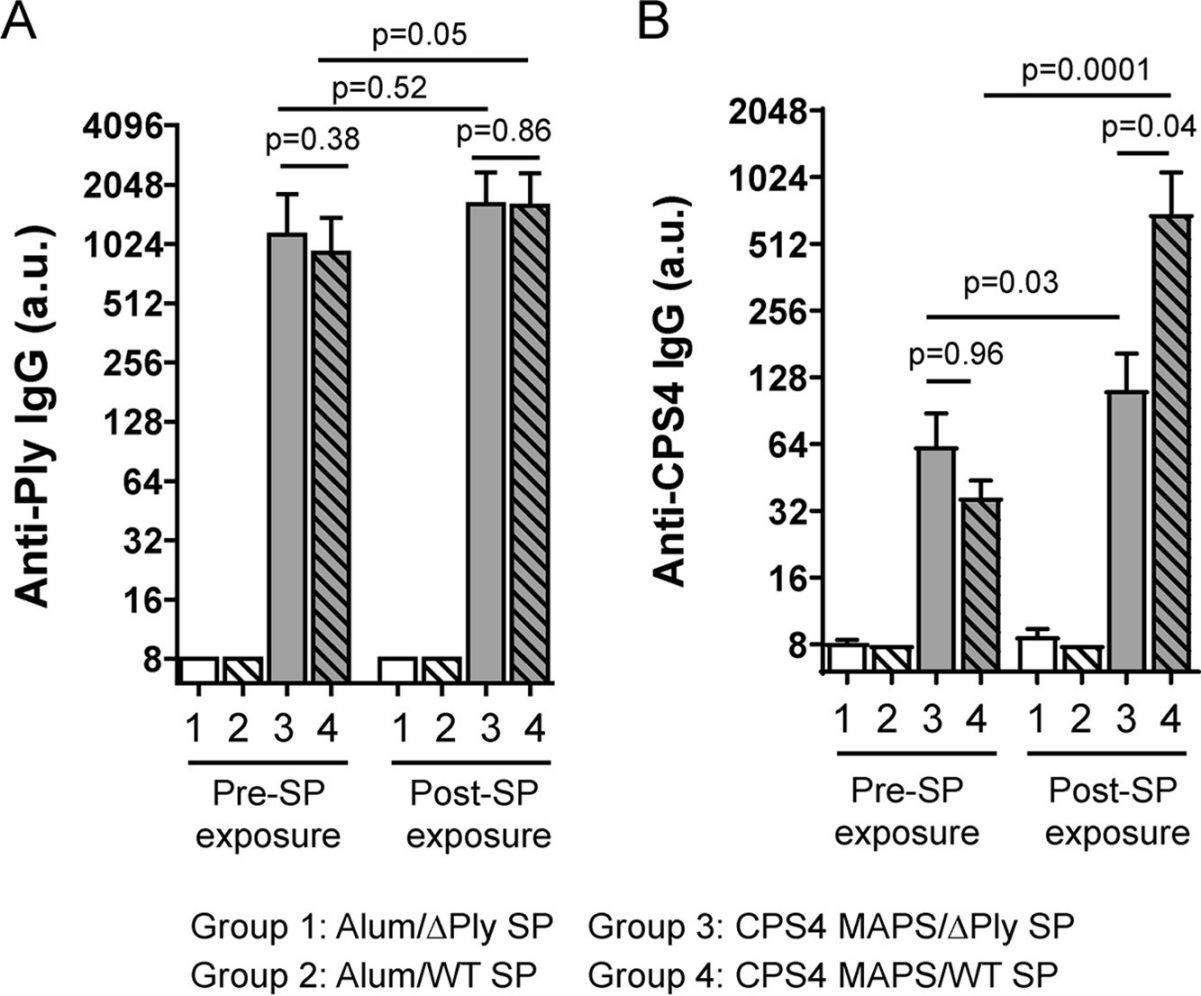
The use of a pathogen-homologous carrier protein enhances anti-CPS IgG production in vaccinated mice during pneumococcal exposure. C57BL/6 mice (*n* = 10 for groups 1 and 2, and *n* = 15 for groups 3 and 4) received one immunization with adjuvant (alum) alone (groups 1 and 2) or adjuvanted CPS4 MAPS vaccine (groups 3 and 4) (2 μg of CPS content per mouse). Serum samples were collected 14 days after immunization (Pre-SP exposure). Two weeks after the bleed, mice were exposed to either the heat-killed wild-type (WT) (groups 1 and 3) or pneumolysin knockout (ΔPly) (groups 2 and 4) Tigr4 strain, each at 1 μg of CPS4 content (2.5 or 2.1 μg of protein content for the WT or ΔPly, respectively) per mouse via intraperitoneal injection. Serum samples were collected 14 days after exposure (Post-SP exposure). Anti-Ply (A) and anti-CPS IgG antibodies (B) were measured using an ELISA. Antibody titers are expressed in arbitrary units (a.u.) relative to a reference serum sample of Ply or CPS4 antigen. Bars represent geometric means and 95% CIs. Statistical analyses were performed using the Mann-Whitney U test between the indicated groups.

To test this hypothesis, we prepared a MAPS vaccine by coupling CPS4 with a fusion protein consisting of rhizavidin and PdT, a toxoid of pneumolysin (Ply) (a pneumococcal cytolysin [27]). Following one immunization with CPS4 MAPS, mice developed a strong antibody response to PdT and a low level of anti-CPS4 IgG (Fig. 6A and B, groups 3 and 4, Pre-SP exposure). Four weeks later, mice were intraperitoneally exposed to either heat-killed WT serotype 4 pneumococci or a heat-killed isogenic Ply knockout strain (ΔPly) (Fig. S4); importantly, both killed bacterial preparations were normalized to have the same amount of CPS4 injected into mice. When evaluated 2 weeks later, mice exposed to the killed WT strain produced a significantly larger amount of anti-CPS4 IgG than mice exposed to the killed ΔPly strain (Fig. 6, groups 3 and 4, Post-SP exposure), confirming our hypothesis that prior exposure to the toxoid via immunization (to generate Ply-specific memory Th cells) would enhance the recall anti-CPS response to Ply-containing pneumococci.

10.1128/mbio.03790-21.5 FIG S4 Western blotting of the heat-killed wild-type or pneumolysin knockout Tigr4 strain. Heat-killed wild-type (WT) or pneumolysin (Ply) knockout (ΔPly) Tigr4 pneumococci were loaded onto SDS-PAGE gels (∼30 μg of total protein content per sample) and then transferred to a polyvinylidene difluoride (PVDF) membrane for Western blotting using rabbit immune sera against Ply or against SP0785 or SP1500 (as positive controls). Compared to the WT strain, the ΔPly strain showed no Ply expression but normal expression of SP0785 or SP1500. Download FIG S4, TIF file, 1.0 MB. Copyright © 2022 Zhang et al. 2022 Zhang et al. https://creativecommons.org/licenses/by/4.0/ This content is distributed under the terms of the Creative Commons Attribution 4.0 International license.

## DISCUSSION

CPSs are attractive targets of bacterial vaccines. As one of the most abundant molecules on the surface of encapsulated bacteria, CPSs are highly accessible to antibodies, which can promote opsonophagocytic killing of the organism. At the same time, the TI nature of CPS limits antibody responses during natural exposure or following immunization with purified, uncoupled CPS molecules. Conjugate vaccines have circumvented this issue, but the mechanism explaining how the carrier protein may mediate the enhanced immunogenicity of the CPS has not been fully elucidated. As a result, the approach used to construct conjugate vaccines has not changed much over the subsequent 3 decades, and the selection of carrier proteins for use in new conjugate vaccines has been guided primarily by previous clinical experience rather than new data.

In a previously proposed model for conjugate vaccines, the major function of carrier proteins is to help implement MHCII presentation for the attached CPS and thus lead to the activation of B_CPS_ via the interaction with cognate T_CPS_ (15). In this framework, the covalent linkage between CPS and carrier proteins is essential for both the priming and recall of anti-CPS antibody responses during vaccination. Our study here, using the MAPS technology (in which CPS and carrier proteins are coupled via strong-affinity interactions rather than covalent bonds of conjugate vaccines), both confirms and extends this model.

We demonstrate that carrier proteins facilitate the generation of anti-CPS immunity in three stages. First, the association with carrier proteins modifies the interaction of CPS with APCs. Previously, it was believed that the poor immunogenicity of CPSs is due to their inability to interact with MHCII molecules and, thus, prime Th cells. Our results suggest that the failure may happen even prior to presentation: we show that purified CPS molecules have low binding and internalization in APCs, thus limiting the possibility of surface presentation. We also show that either a direct (as in MAPS) or an indirect (as in bacterial cells) association of CPS with proteins could significantly enhance the binding, uptake, processing, and, ultimately, surface presentation of CPS molecules in APCs. However, we found that only a direct/strong connection between CPS and carrier proteins, like the biotin-avidin/rhavi interaction (*K_d_* of ∼10^−15^ M; low-pH and protease digestion resistant) (19, 28) (see Fig. S5 in the supplemental material) in MAPS or, as shown previously, the covalent bond in conjugate vaccines, will lead to MHCII-dependent presentation of the internalized CPS. When CPS is not directly associated with proteins, as in bacterial cells, the internalized CPS can also be presented but in an MHCII-independent manner (e.g., via CD1d), which may lead to the activation of another type(s) of T cell, such as NKT cells, as described previously (16, 25, 29). As the interaction was evaluated by an inhibition ELISA, some technical limitations of our experiments should be noted. First, the amount of surface CPS was evaluated by subtracting intracellular CPS from the total cell-associated CPS. This indirect approach may introduce some variability into the results but importantly avoids the need to fix cells, which may disrupt the surface structure of cells and, thus, invalidate the results. Second, the polyclonal rabbit sera used for the inhibition ELISA were not specifically characterized for individual antigenic epitopes of the target CPS, which could influence the sensitivity of the detection of processed sugars on the cell surface associated with the carrier/MHCII. Finally, the significant increase of surface CPS at 37°C could hypothetically be explained by the enhanced binding of MAPS complexes at physiological temperature. However, we do not believe that this is likely to be the case since such temperature-dependent binding is generally a rapid process and should reach equilibrium within 0.5 to 1 h of incubation. The fact that there was a large increase in the detection of surface CPS even between the 6-h and 18-h incubations at 37°C (from 0.6 μg to 1.6 μg) suggests that most of this increase was likely due to the surface presentation of processed CPS rather than the binding of MAPS complexes.

Next, we demonstrate that the TD activation of naive B_CPS_ can be mediated by two separate populations of Th cells, T_CPS_ or T_carrier_. T_CPS_-mediated B_CPS_ activation was previously shown with conjugate vaccines (15, 30, 31). Here, we confirmed this mechanism with MAPS vaccines made of different CPS antigens. Moreover, we demonstrate that in addition to T_CPS_, T_carrier_ can also facilitate the activation of B_CPS_. We further show that these two activation mechanisms do not apply equally for all CPS antigens: T_CPS_-mediated activation appears to work for a broader range of CPS antigens, whereas T_carrier_-mediated activation applies to some CPSs but not others.

10.1128/mbio.03790-21.6 FIG S5 The avidin (Avi) protein in the MAPS complex is more resistant to proteinase digestion and remains bound to biotinylated CPS even after being partially digested. The MAPS complex was made with biotinylated CPS14 and avidin. For *in vitro* digestion, each sample contains 2 μg of avidin protein in the form of a MAPS complex or as free protein. Samples were incubated with or without proteinase K (0.5 mg/mL) at 37°C for 8 h and then treated with reduced SDS sample buffer at room temperature or with boiling for 10 min before being applied onto an SDS-PAGE gel. For MAPS samples, the avidin-biotin interaction is stable in the presence of SDS at room temperature, and thus, avidin protein remains in the loading well of the gel due to the large size of biotinylated CPS14 (line 1, red arrow). After boiling, the avidin protein is dissociated from biotinylated CPS14 and runs into the gel (line 2). For free protein samples, avidin runs into the gel with or without boiling (lines 5 and 6). After protease K treatment, about 73% (the intensity of the Avi band in line 4 compared to that of the Avi band in line 2) of the avidin proteins in the MAPS complex remain intact (line 4, Avi), and the rest are partially digested (line 4, Avi F). Nevertheless, the partially digested avidin can still bind to biotinylated CPS14 if the sample is kept at room temperature (line 3, red arrow). In contrast, after the same protease K treatment, only 6% (the intensity of the Avi band in line 8 compared to that of the Avi band in line 6) of biotin-free avidin was left undigested (line 8, Avi). Download FIG S5, TIF file, 2.0 MB. Copyright © 2022 Zhang et al. 2022 Zhang et al. https://creativecommons.org/licenses/by/4.0/ This content is distributed under the terms of the Creative Commons Attribution 4.0 International license.

Finally, we demonstrate an important role of memory Th cells, especially memory T_carrier_, in enhancing recall anti-CPS responses. We first confirm that recall anti-CPS responses mediated by memory B_CPS_ can be induced in a TI manner (either by pure CPS or by MAPS complexes in the absence of T cells). This finding is consistent with clinical observations that anti-CPS responses can be boosted following exposure to pure CPS in toddlers who have been previously primed with conjugate vaccines. Next, we elucidate the important difference between the TI and TD recall responses: with TD recall responses, the level of anti-CPS antibody production by memory B_CPS_ can be significantly enhanced compared to that following TI activation. We also demonstrate that such an enhancement is primarily mediated by memory rather than naive Th (T_CPS_ and T_carrier_) cells. This finding provides a strategy to potentiate recall anti-CPS responses in vaccinated individuals during infections by using pathogen-homologous proteins as carriers of CPS vaccines. Immunization with such vaccines can induce memory T_carrier_ that can be activated upon exposure to the bacterial protein and thus mediate enhanced, TD activation of memory B_CPS_ during infections, as illustrated here using a mouse model of pneumococcal infection.

In conclusion, our work demonstrates the diverse roles of carrier proteins in facilitating T cell-mediated anti-CPS responses and reveals a dual TD activation mechanism of B_CPS_ during priming and recall responses. The incorporation of pathogen-specific antigens as carriers may provide additional boosting following exposure to the pathogen. Overall, our findings thus provide new insights into immunological responses to CPS, which can guide the selection of carrier proteins for the development of more effective CPS vaccines.

## MATERIALS AND METHODS

### Mouse strains.

Wild-type, MHCII^−/−^, and Rag1^−/−^ C57BL/6 mice (female, 5 to 6 weeks of age) were all purchased from Jackson Laboratories.

### Ethics statement.

All procedures involving mice were approved by the Boston Children’s Hospital animal care and use committee (IACUC protocol number 19-10-4051R), according to National Institutes of Health guidelines for animal housing and care (34).

### Cloning and protein purification.

DNA sequences encoding SP1500 (amino acids 27 to 278) or SP0785 (amino acids 33 to 399) were amplified from S. pneumoniae genomic DNA (purified from the Tigr4 strain) via PCR. The DNA sequence encoding pneumolysin toxoid (PdT) was obtained by PCR from the pQE-30-PdT plasmid (20). Purified PCR products were then cloned into a pET-21b vector for the recombinant expression of C-terminally His_6_-tagged proteins. For rhizavidin fusion proteins, DNA sequences encoding the indicated pneumococcal proteins were inserted at the 3′ end of the gene encoding the rhizavidin moiety in a pET-21b vector (20). All constructs were transformed into the Escherichia coli BL21(DE3) strain for expression. His-tagged recombinant proteins were purified using nitrilotriacetic acid (NTA) affinity chromatography (Qiagen) followed by size exclusion chromatography using a Superdex 200 column (Cytiva). The protein concentration was determined using the bicinchoninic acid (BCA) protein assay kit (Thermo Scientific). Purified proteins were stored at −80°C until use.

### Preparation of MAPS complexes.

Avidin was purchased from Sigma-Aldrich. Pneumococcal CPSs were purchased from the ATCC. Biotinylation of CPS was done as described previously, using 1-cyano-4-dimethylaminopyridinium tetrafluoroborate (CDAP) as the activation reagent (20). MAPS complexes were prepared by the incubation of biotinylated CPS with the indicated carrier proteins at room temperature overnight, with an input ratio of protein to CPS of 3:1 (wt/wt). Assembled MAPS complexes were purified by size exclusion chromatography. CPS and protein concentrations of purified MAPS complexes were measured by an anthrone assay (32) and a BCA protein assay kit, respectively. Purified MAPS complex was stored at 4°C in the presence of 0.01% thimerosal until use.

### Immunizations and antiserum production.

All vaccines were prepared 1 day before immunization. Antigens were diluted to the appropriate concentration in saline, mixed with aluminum phosphate (Brenntag) (1.25-mg/mL final concentration of aluminum content), and then incubated at 4°C overnight with rotation (24 rpm). Mice received subcutaneous immunization with the indicated vaccine in 200 μL, up to three times, 2 weeks apart. Sera were collected 2 weeks after each immunization or as indicated for antibody analysis.

For rabbit immune serum generation, New Zealand White rabbits (*n* = 2 per group) received three intramuscular immunizations with CPS14-carrier 1 or CPS4-carrier 1 MAPS (1 μg of CPS content per dose), 2 weeks apart. Sera were collected 2 weeks after the last immunization and analyzed by an ELISA against CPS14 or CPS4. The serum that had the highest CPS-specific IgG antibody titer was used for an inhibition ELISA.

### Adoptive cell transfer.

Donor mice were immunized once with the indicated MAPS complex (at 1 μg of each CPS dosage) or carrier protein (10 μg) and then housed for at least 2 months before the collection of splenocytes. For splenocyte preparation, donor mice were euthanized, and spleens were dissected and processed as described previously (33). B cells or CD4^+^ T cells were further purified from splenocytes using CD19 or CD4 MicroBeads (Miltenyi Biotec) according to the manufacturer’s instructions. All cell preparations were then resuspended in phosphate-buffered saline (PBS) with 2% bovine serum albumin (BSA). For adoptive transfer, each Rag1^−/−^ mouse received an intraperitoneal injection with 2.5 × 10^7^ B cells, with or without an additional 1.25 × 10^7^ CD4^+^ T cells, as indicated.

### Statistical analysis.

All statistical analyses were done using Prism (version 5.01 for Windows; GraphPad Software, Inc.). Differences between groups were compared using a nonparametric, two-tailed Mann-Whitney U test.

10.1128/mbio.03790-21.1 TEXT S1 Additional materials and methods. Details of binding, internalization, and antigen presentation assays are described, as are methods of bacterial preparation, antibody analysis, and inhibition ELISAs. Download Text S1, DOCX file, 0.02 MB. Copyright © 2022 Zhang et al. 2022 Zhang et al. https://creativecommons.org/licenses/by/4.0/ This content is distributed under the terms of the Creative Commons Attribution 4.0 International license.
