# Supplementary figures and images for: Carrier Proteins Facilitate the Generation of Antipolysaccharide Immunity via Multiple Mechanisms
Source: mBio. 2022 Apr 14;13(3):e03790-21. doi: 10.1128/mbio.03790-21 (PMC9239039; doi:10.1128/mbio.03790-21)

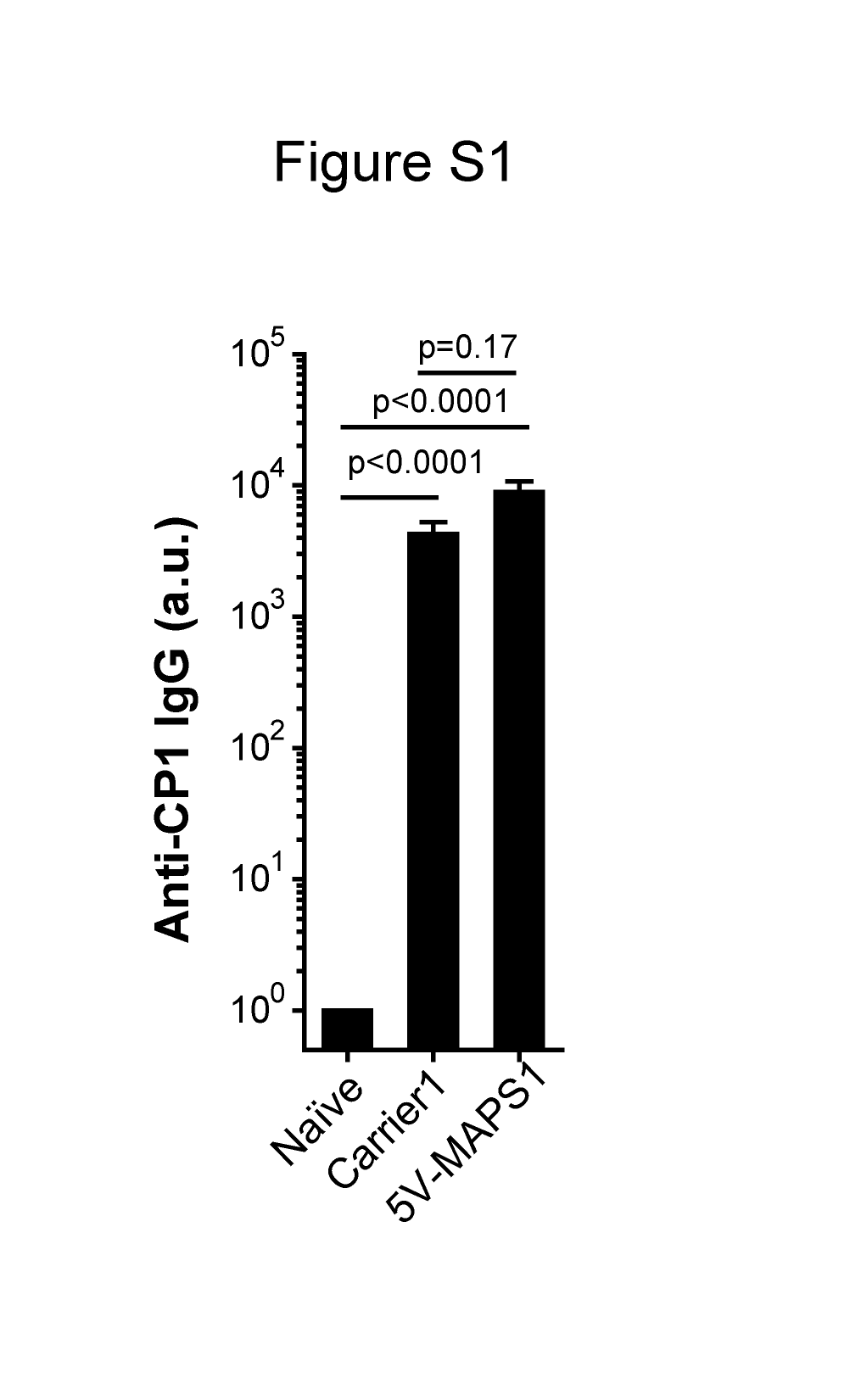

Supplement: FIG S1 [file mbio.03790-21-s0002.tif]

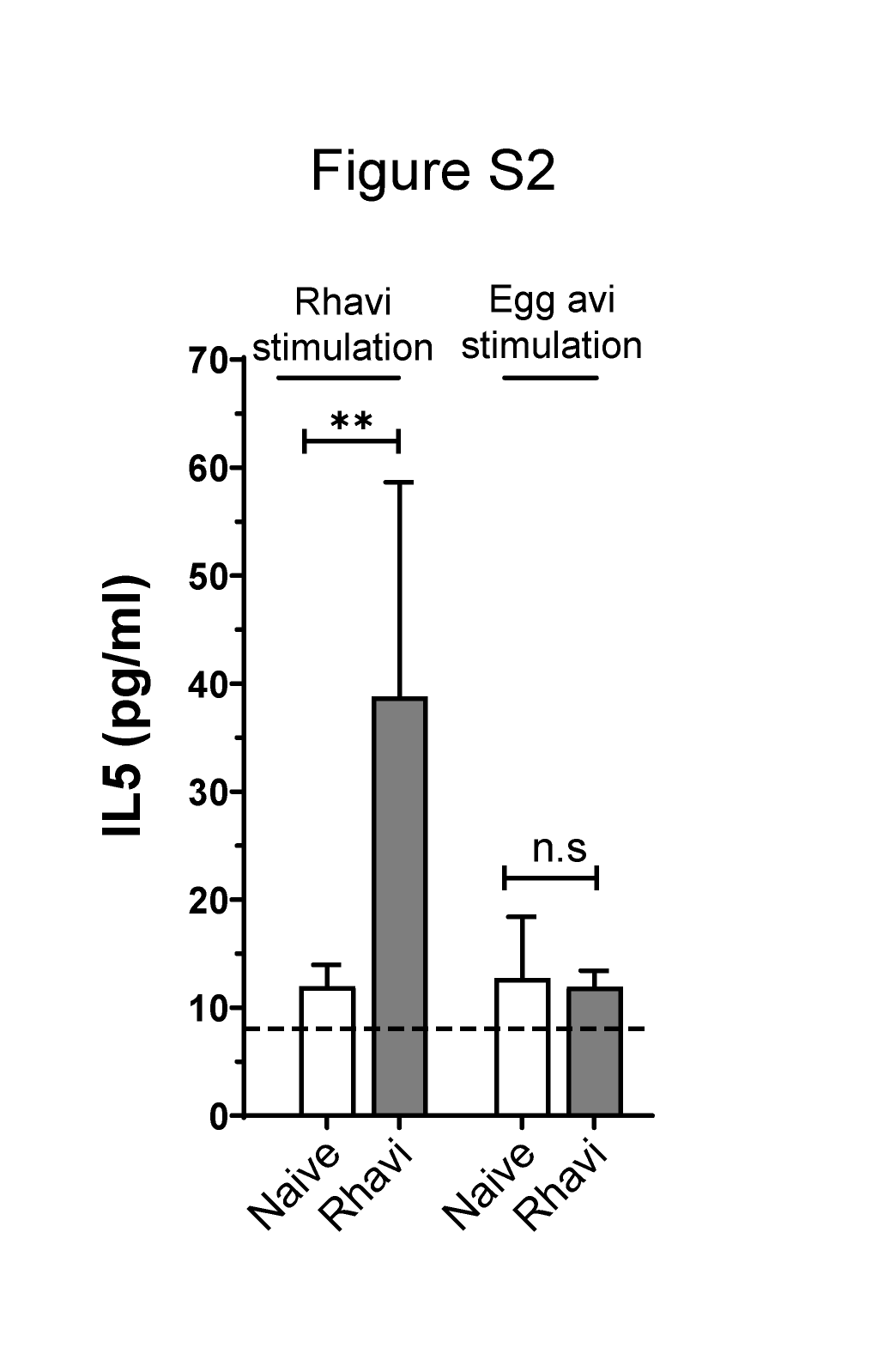

Supplement: FIG S2 [file mbio.03790-21-s0003.tif]

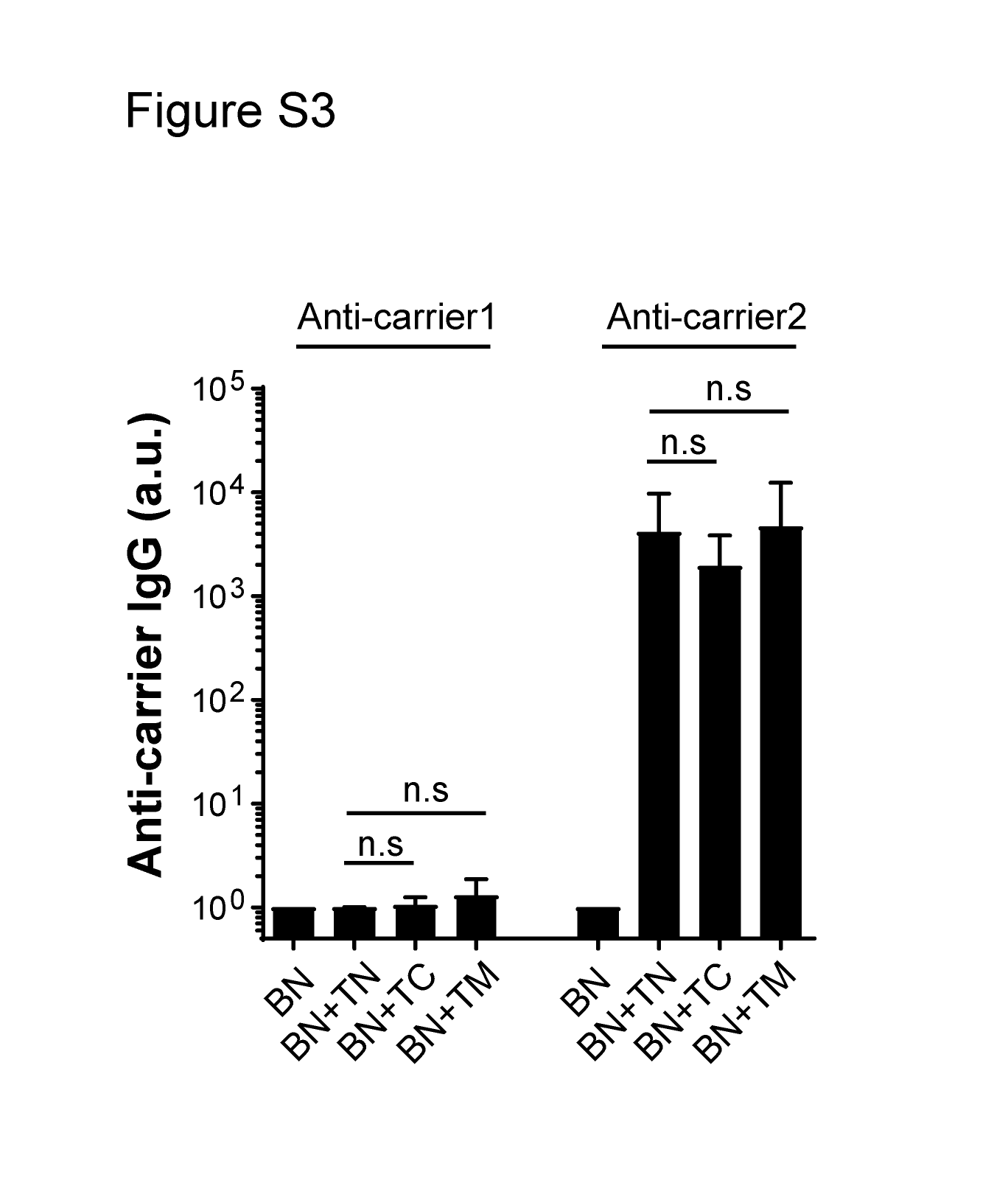

Supplement: FIG S3 [file mbio.03790-21-s0004.tif]

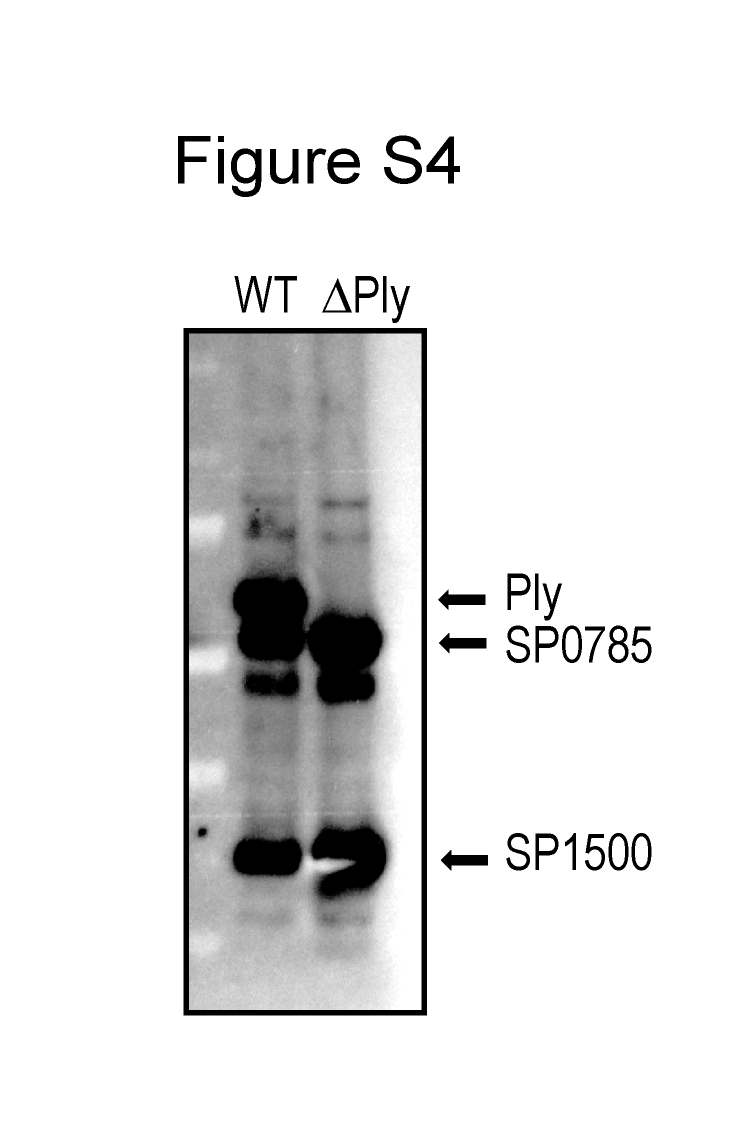

Supplement: FIG S4 [file mbio.03790-21-s0005.tif]

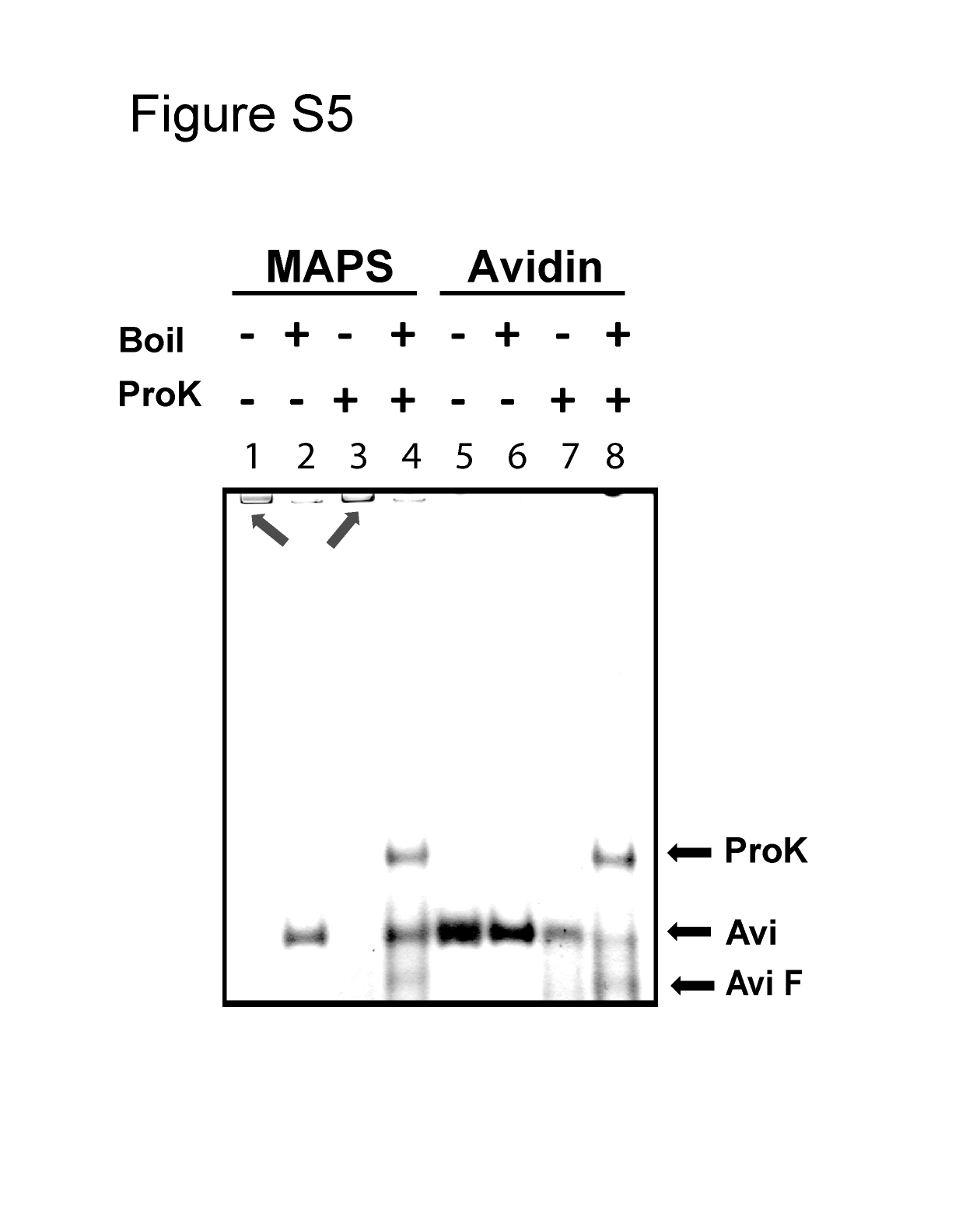

Supplement: FIG S5 [file mbio.03790-21-s0006.tif]
