# Supplementary material for: Carrier Proteins Facilitate the Generation of Antipolysaccharide Immunity via Multiple Mechanisms
Source: mBio. 2022 Apr 14;13(3):e03790-21. doi: 10.1128/mbio.03790-21 (PMC9239039; doi:10.1128/mbio.03790-21)
Supplement: TEXT S1 [file mbio.03790-21-s0001.docx]

**Binding, internalization, and presentation of CPS antigens in macrophages.** Peritoneal macrophages were isolated from C57BL/6 mice after intraperitoneal injection with 3% Brewer thioglycollate medium (3 mL per mouse) and stimulation for 3 days. To collect the macrophages, mice were euthanized, the abdominal skin was retracted, and 10 mL of cold PBS was injected along the left side of the peritoneal wall. The cell suspension was then aspirated using a syringe and dispensed into a 50-mL conical tube on ice. Cells were pelleted by centrifugation, resuspended in culture medium (DMEM/F12 medium with 10% FBS) and plated in 12-well plates at 1×10^6^ cells per mL per well. Unattached cells were removed after 3-hour incubation at 37 °C, and 0.5 mL of fresh medium containing plain CPS14, MAPS complex or heat-killed bacteria (all at 2.5 μg/mL of CPS content) was added to each well for incubation at 4 °C or 37 °C for the indicated time (4 wells per condition). After incubation, the medium was discarded, and cells were washed with PBS twice before further treatment for CPS analysis.

For CPS quantification, we measured total cell-associated CPS and intracellular CPS content at each condition by inhibition ELISA and then calculated surface-associated CPS based on the difference between these two measurements. For every 6 wells of cells treated at the same condition, 3 wells were incubated with 1:200 dilution of pre-immune rabbit sera (for measurement of the total cell-associated CPS) and 3 wells were incubated with 1:200 dilution of rabbit anti-CPS14 sera (to block surface-associated CPS for measurement of the intracellular CPS content) for 30 minutes RT. After incubation, cells were washed PBS three times and then incubated with cold water containing 2 mM EDTA and proteinase inhibitor cocktail (Thermo Scientific) (150 μL per well) at 4 °C for 1 hour. Cells were then detached from the plates by pipetting, and then transferred into PCR tubes and lysed by sonication in an ice-water bath. At the end of sonication, 15 μL of 10× PBS concentrate was added into each sample to adjust the pH to 7.5. CPS content in each sample was measured by inhibition ELISA and then normalized to the concentration of total cellular proteins measured by the BCA protein assay kit.

For Western blot and Co-IP, 5×10^6^ peritoneal macrophages were seeded in a 60 mm dish with 5 mL culture medium. Unattached cells were removed after 3-hour incubation at 37 °C, and 3 mL of fresh medium containing 2.5 μg/mL of CPS14 or MAPS complex (in CPS content), or 7.5 μg/mL of avidin protein was added. After 18-hour incubation at 37 °C, the medium was discarded, and cells were washed with PBS twice, detached from the dish and lysed in 250 μL of cold lysis buffer (20 mM Tris, pH7.5, 150 mM NaCl, 2mM EDTA, 0.1% Triton X-100 and proteinase inhibitor cocktail) by sonication. For Co-IP, cell lysates were mixed with protein A resin (GE Healthcare Life Science) that had been pre-treated with rabbit anti-CPS14 serum and then washed extensively with PBS for overnight incubation at 4 °C. After incubation, the resin was pelleted, washed with lysis buffer extensively and then boiled in 30 μl of reduced SDS sample buffer. Cell lysates and Co-IP samples were applied to SDS-PAGE and then transferred onto PVDF membrane for Western blot using rabbit anti-avidin sera, rabbit monoclonal antibody against β-actin (Abcam) and rabbit polyclonal antibody against MHC-II (Abcam), as primary antibodies followed by HRP-conjugated anti-rabbit secondary antibody. The membrane was developed using ECL substrate (Thermo Scientific) and exposed on film.

**Preparation of heat-killed pneumococci.** Pneumococcal strain 1401 (serotype 14) and Tigr4 (serotype 4) were used in this study. A pneumolysin knockout Tigr4 strain was constructed using the procedure described previously (1, 2). Bacteria were grown in Todd-Hewitt medium plus 0.5% yeast extract (THY) at 37°C with 5% CO_2_ to OD_600_=0.05, and the transformation was done as described previously (3). Transformants were selected on Trypticase soy agar plates with 5% sheep blood (BAP), supplemented with 400 μg/mL Kanamycin and 600 μg/mL gentamicin. Mutants were confirmed by PCR and sequencing the genomic region of pneumolysin, as well as by Western blot probing for the pneumolysin protein.

To prepare heat-killed pneumococci, pneumococcal strains were streaked onto BAP and grown overnight at 37°C with 5% CO_2_. The overnight culture was collected in the morning and inoculated into THY medium and grown at 37°C with 5% CO_2_ until OD_600_=0.8. Bacteria were then pelleted by centrifugation, washed twice with PBS, resuspended in PBS (to OD_600_=4) and heat-inactivated by incubation at 58°C for 1 hour. After inactivation, a sample of bacteria was plated on BAP and cultured overnight at 37°C with 5% CO_2_ for confirmation of a complete killing. Protein and CPS concentrations of heat-killed pneumococci were determined by BCA assay and inhibition ELISA, respectively.

**Antibody analysis.** Antigen-specific antibodies were measured by ELISA. For CPS antigens, Immulon 2 HB 96-microwell plates (Thermo Scientific) were coated with CPS solution (0.5-5 μg/mL in PBS) for 5 hours at 37 °C and then overnight at 4 °C. For proteins, plates were coated with indicated protein (1 μg/mL in PBS) overnight at room temperature. Coated plates were washed with PBS containing 0.05% Tween 20 (PBST) and then blocked with 1% BSA in PBS for 1 hour. After blocking, serial dilutions of the reference serum and sample sera were added and incubated for 2 hours, followed by a 1-hour incubation with HRP-conjugated secondary antibody against mouse IgM or IgG. The plates were then washed and developed with SureBlue TMB Microwell Peroxidase Substrate (KPL). 1M HCl was used to terminate the reactions before the A_450nm_ was analyzed using an ELISA reader. Antibody titers were expressed in arbitrary units relative to the reference serum (generated by pooling sera from mice that have been immunized three times with 5V MAPS1 or with indicated carrier proteins). Antigen-specific IgM or IgG titer of each reference serum was arbitrarily assigned as 12,000 units/mL.

IgG avidity was measured using the method described previously (4). Briefly, serum samples were diluted with PBST to the appropriate concentration and then added to CPS14-coated microplates (8 wells per sample, 100 μL per well) for a 2-hour incubation. After washing with PBST, seven 2-fold serial dilutions of sodium thiocyanate (NaSCN) (4M, in H_2_O) or a blank (H_2_O only) were added to the wells of each sample (100 μL per well) and incubated for 30 min. Plates were then washed, incubated with HRP-conjugated secondary antibody for 1 hour, and washed again before development with SureBlue. Avidity was expressed as the avidity index (A.I.), the molar concentration of NaSCN that elutes 50% of CPS14-specific IgG antibodies that bind onto the plates.

**Inhibition ELISA for CPS quantification.** CPS content in heat-killed pneumococci or different cellular preparations was measured by inhibition ELISA using polyclonal rabbit sera against CPS14 or CPS4. 96-well microplate was coated with CPS14 or CPS4 and then blocked with 1% BSA in PBS. In a separate assay microplate (not treated for ELISA antigen coating), in each well, 50 μL of samples or purified CPS (at different concentrations, as standard reference) were mixed with 50 μL of rabbit anti-CPS14 sera (1:1600 dilution) or anti-CPS4 sera (1:800 dilution), incubated for 30 min, and then the mixture was transferred into the ELISA plate for an additional incubation of 2 hours. The plate was then washed, incubated with an HRP-conjugated secondary antibody and developed as described above for regular ELISA. The concentration of CPS in each sample was calculated according to the standard reference.

1. Hua CZ, Howard A, Malley R, Lu YJ. 2014. Effect of nonheme iron-containing ferritin Dpr in the stress response and virulence of pneumococci. Infect Immun 82:3939-47.

2. Sung CK, Li H, Claverys JP, Morrison DA. 2001. An rpsL cassette, janus, for gene replacement through negative selection in Streptococcus pneumoniae. Appl Environ Microbiol 67:5190-6.

3. Bricker AL, Camilli A. 1999. Transformation of a type 4 encapsulated strain of Streptococcus pneumoniae. FEMS Microbiol Lett 172:131-5.

4. Colino J, Duke L, Snapper CM. 2013. Noncovalent association of protein and capsular polysaccharide on bacteria-sized latex beads as a model for polysaccharide-specific humoral immunity to intact gram-positive extracellular bacteria. J Immunol 191:3254-63.
